# Supplementary material for: Irradiation Mediates IFNα and CXCL9 Expression in Non-Small Cell Lung Cancer to Stimulate CD8+ T Cells Activity and Migration toward Tumors
Source: Biomedicines. 2021 Sep 29;9(10):1349. doi: 10.3390/biomedicines9101349 (PMC8533192; doi:10.3390/biomedicines9101349)
Supplement: Supplementary file 1 [file biomedicines-09-01349-s001.zip › biomedicines-1391862-supplementary.pdf]

**Table S1. Distribution of T cell subpopulations in the enrolled volunteers**

| Sample number | CD4$^+$ | | | | | | |
|---|---|---|---|---|---|---|---|
| | CD16$^+$ (%) | CD25$^+$ (%) | CD28$^+$ (%) | CD122$^+$ (%) | CD178$^+$ (%) | CD183$^+$ (%) | CD279$^+$ (%) |
| N1 | 16.7 | 18.7 | 58.6 | 82.6 | 16.8 | 51 | 87.8 |
| N2 | 12.9 | 10.5 | 43.4 | 87.4 | 14.7 | 38.2 | 93.7 |
| N3 | 15.8 | 18.9 | 68.5 | 53.2 | 14.3 | 49.9 | 27.1 |
| N4 | 14.7 | 17.2 | 60 | 51.1 | 10.8 | 39.3 | 18.8 |
| P1 | 11.6 | 18.7 | 55.6 | 88.7 | 13.8 | 48 | 82.2 |
| P2 | 14.6 | 16.8 | 48.6 | 90.3 | 15.3 | 43.4 | 69.8 |
| P3 | 24.6 | 14.7 | 38 | 86.6 | 14.3 | 56 | 99.5 |
| P4 | 18.4 | 20.9 | 62.3 | 59.5 | 8.94 | 43.9 | 39.1 |
| Sample number | CD8$^+$ | | | | | | |
| | CD16$^+$ (%) | CD25$^+$ (%) | CD28$^+$ (%) | CD122$^+$ (%) | CD178$^+$ (%) | CD183$^+$ (%) | CD279$^+$ (%) |
| N1 | 15.7 | 9.83 | 39.1 | 90.5 | 16.1 | 71.9 | 82 |
| N2 | 28.1 | 8.77 | 22.7 | 93.1 | 14.8 | 50.1 | 95 |
| N3 | 31.9 | 11.9 | 29.7 | 76.6 | 15.3 | 88.4 | 58 |
| N4 | 18.1 | 11.6 | 34.6 | 67.2 | 12.2 | 80 | 38 |
| P1 | 42.6 | 12.1 | 23.8 | 96.3 | 13.4 | 61.6 | 90.2 |
| P2 | 17.6 | 11.3 | 29.2 | 97.2 | 15.9 | 48.6 | 92.3 |
| P3 | 33.1 | 7.93 | 17.4 | 96.3 | 14.3 | 71.8 | 90.5 |
| P4 | 29.7 | 7.89 | 27.2 | 93.1 | 9.39 | 63.4 | 84.7 |

Protein name, *CD25*: IL2Rα; *CD122*: IL2Rβ; *CD178*: FasL; *CD183*: CXCR3; *CD279*: PD-1.

**Table S 2. Detailed operations for the experiments**

**Isolation of PBMCs and CD8$^+$ T cells:**

Blood was collected from the individual donors in Mackay Memorial Hospital, Taipei, Taiwan (*n* = 11, Table 1). In brief, 3 mL of buffy coats were isolated from 10 mL of whole blood by an initial 1200 rpm centrifugation for 30 min without braking. The collected 3 mL of buffy coats was mixed with 4 mL of PBS buffer and loaded onto the 4 mL of ficoll solution and consequently for 2000 rpm gradient centrifugation for 20 min at 18-20ºC. PBMCs on the interface between the plasma and ficoll media were collected and cryopreserved in FBS containing 10% DMSO at -80 ºC for further analysis. The CD8$^+$ T cell and Non-CD8$^+$ PBMCs were isolated using a MACS separation isolation kit (Miltenyi Biotec, North Rhine-Westphalia, Germany) by following the manufacturer's manual.

***In vitro* CD8$^+$ T cell migration:**

In brief, $1 \times 10^4$ A549 cells and $9 \times 10^5$ non-CD8$^+$ PBMCs in 750μL of RPMI culture medium were loaded into a 24-well culture plate. Then, $1 \times 10^5$ purified CD8$^+$ T cells labeled with Alexa488-CD8 antibody were placed in the upper layer of a cell culture insert with 150 μL of RPMI culture medium. Cells were incubated at 37 °C and 5% CO2 for 24 h. The migrated cells that had through the membrane filter were determined by a SpectraMax iD3 Multi-Mode Microplate Reader (Molecular Devices, San Jose, CA, USA) for detecting the fluorescence intensity.

***In vivo* PD-1 nuclear imaging:**

Nivolumab, an anti-PD-1 antibody, was conjugated with P-SCN-Bn-DTPA (w/w 1:10, Macrocyclics, Dallas, TX, USA) in carbonate-bicarbonate buffer (pH 9.0) at room temperature for 2 h. The DTPA-conjugated nivolumab was purified using a G-25 column, whereas the second ml was collected. The labeling ratio of antibodies with DTPA was analyzed using a matrix-assisted laser desorption/ionization time-of-flight mass spectrometer (MALDI-TOF-MS, UltraflexIII, Bruker Daltonics GmbH, Germany). The 10 μg of nivolumab-DTPA was labeled with 10 mCi of $^{111}$In for 2 hours and the labeled rate > 80% was acceptable for consequent used in the tumor xenografts. The labeling efficiency was measured using an instant thin layer chromatography (iTLC) on the silica gel impregnated glass fiber sheets (PALL Corporation, USA), whereas PBS was used as the mobile phase. Then, the sheets were measured using a radioactive scanner (AR-2000radio-TLC Imaging Scanner, Bioscan, France). The tumor xenografts were intravenously injected with $^{111}$In-nivolumab (n=3) by 100 μCi of radioactivity for each mouse. A Nano-SPECT/CT (Mediso Medical Imaging Systems, USA) was utilized to detect and image the tumors in the tumor model *in vivo*. To quantify the radioactive tumor, 8 μCi calibrated standard was used in every imaging experiment, whereas the region of intensity in tumor tissues was calculated based on the standard. The radioactive images were captured post 3, 24, 48 hours after $^{111}$In-labeled nivolumab injection.

**ELISA for CXCL9 detection:**

In brief, A549 cells with or without 20 Gy treatment were cultured for 24 hours at 37 °C overnight. The culture medium was collected and clarified by 2000 rpm centrifugation for 10 min and each 100 μl of the supernatant medium was added to pre-coated anti-CXCL9 antibody in a 96-well microplate. For detecting CXCL9 concentration in the serum between healthy volunteers and patients with lung cancer, individual 50 μl of serum was used and pre-coated with the anti-CXCL9 antibody in a 96-well

microplate. The consequent steps, including washing, secondary antibodies incubation, and fluorescence detection are completed according to the manufacturer’s instruction.
